# Whole genome sequencing of nearly isogenic WMI and WLI inbred rats identifies genes potentially involved in depression and stress reactivity

**DOI:** 10.1038/s41598-021-92993-4

**Published:** 2021-07-20

**Authors:** Tristan V. de Jong, Panjun Kim, Victor Guryev, Megan K. Mulligan, Robert W. Williams, Eva E. Redei, Hao Chen

**Affiliations:** 1 University of Tennessee Health Science Center, Memphis, TN USA; 2 European Research Institute for the Biology of Ageing, University of Groningen, Groningen, The Netherlands; 3 Northwestern University – Chicago, Chicago, IL USA

**Keywords:** Behavioural genetics, Genetics, Next-generation sequencing

## Abstract

The WMI and WLI inbred *rats* were generated from the stress-prone, and not yet fully inbred, Wistar Kyoto (WKY) strain. These were selected using bi-directional selection for immobility in the forced swim test and were then sib-mated for over 38 generations. Despite the low level of genetic diversity among WKY progenitors, the WMI substrain is significantly more vulnerable to stress relative to the counter-selected WLI strain. Here we quantify numbers and classes of genomic sequence variants distinguishing these substrains with the long term goal of uncovering functional and behavioral polymorphism that modulate sensitivity to stress and depression-like phenotypes. DNA from WLI and WMI was sequenced using Illumina xTen, IonTorrent, and 10X Chromium linked-read platforms to obtain a combined coverage of ~ 100X for each strain. We identified 4,296 high quality homozygous SNPs and indels between the WMI and WLI. We detected high impact variants in genes previously implicated in depression (e.g. *Gnat2*), depression-like behavior (e.g. *Prlr*, *Nlrp1a*), other psychiatric disease (e.g. *Pou6f2*, *Kdm5a*, *Reep3*, *Wdfy3*), and responses to psychological stressors (e.g. *Pigr*). High coverage sequencing data confirm that the two substrains are nearly coisogenic. Nonetheless, the small number of sequence variants contributes to numerous well characterized differences including depression-like behavior, stress reactivity, and addiction related phenotypes. These selected substrains are an ideal resource for forward and reverse genetic studies using a reduced complexity cross.

## Introduction

Major depressive disorder (MDD) is a common, debilitating disease that is the leading cause of “years lived with disability” worldwide^1^. Genetic factors play important roles in the etiology of MDD. Heritability of MDD is estimated to be between 28 and 44%^2,3^, although recent estimates are over 50%^4^. Genomic variants contributing to depression have been difficult to identify, but large genome-wide association studies (GWAS)^5^ are starting to identify candidates, including variants near *SIRT1*, *LHPP*^6^*, OLFM4*, *MEF2C*, and *TMEM161B*^7^. Meta-analysis of GWAS based on self-reported depression also identified a larger number of independent and significant loci^8,9^, although relying on self-diagnosis may have reduced the reproducibility of the findings^6^. Even when the MDD diagnosis is not based on self-report, the current diagnostic methods are still comparatively subjective and cannot truly characterize subgroups of this complex disease, which are likely affected by differences in genetics. Thus, identification of sequence variants associated with the disease, and the genetic etiology of MDD, remains largely unsolved.

Compared to the high levels of genetic variations among humans (6 million between any two individuals), well defined animal models can tightly constrain both genomic and environmental variables. Many genetic mapping strategies have been developed for model organisms. For example, the reduced complexity cross (RCC) uses offspring from two genetically similar parents that have divergent phenotypes. The number of segregating variants in an RCC is orders of magnitude smaller than in conventional crosses between highly genetically divergent parents^10^. The reduction in the number of segregating variants in the RCC greatly enhances identification of causal variants and genes^11,12^.

In this report we analyze the genomes of two closed related inbred strains of rats selectively bred from the Wistar Kyoto (WKY) rats. The WKY strain had been developed as the normotensive control for the spontaneously hypertensive rat strain and was distributed to vendors and universities between the 4th and 11th generation of inbreeding^13^. At this early stage, the stock varied widely in behaviors^14^. Genetic heterogeneity of the WKY rats has also been documented^14^. The Redei lab obtained WKY rats from Harlan Laboratories (Madison, WI), where they had been bred for 65 generations. However, it is not known whether the sublines (breeding pairs) Harlan obtained at the beginning of the breeding were maintained as sublines or interbred. The WKY strain has become a well-established model of adult and adolescent depression and comorbid anxiety^15–20^. Its behavior mirrors several symptoms of human MDD and anxiety, including anhedonia, disturbed sleep, a reduced appetite and reduced energy, and the attenuation of depression-like behaviors after treatment with antidepressants^21–26^.

A large variability in behavioral and psychological measurements were noted within the WKY strain^27,28^. The variability of behavior in the forced swim test (FST)—one of the most widely utilized tests for depressive behavior in rodents—motivated the bi-directional selection of the animals based on their level of immobility in the FST^29^. Males and females with the least mobility and lowest climbing scores in the FST were mated, producing the WKY *More Immobile* (WMI) line. Males and females with the highest mobility and highest climbing scores were mated, producing the WKY *Less Immobile* (WLI) line. Those animals showing the most extreme FST behavior within each line were selected for subsequent breeding, specifically avoiding sibling mating until the fifth G generation, when filial F matings were initiated.

Throughout the generations, the WMIs consistently have shown significantly greater immobility behavior in the FST than the WLIs^30^. The sex differences observed in the developmental pattern of MDD and its comorbidity with anxiety parallel differences observed in humans^31^. Maternal characteristics of the WMI after birth show similarities to that of women with postpartum depression^32^. Antidepressant treatments, specifically the tricyclic desipramine and the MAO inhibitor phenelzine, but not fluoxetine, alleviate depression-like behavior of WMIs^29^, and enriched environment in adulthood does the same^33^. Resting state functional connectivity differences between WMIs and WLIs, measured by fMRI, are similar to those found in depressed patients^34,35^. Behavioral and hormonal responsiveness to acute and chronic stress also differ between the strains^33,36^. In humans, posttraumatic stress disorder (PTSD) and alcohol use disorder have high comorbidity with major depression. As hypothesized, the stress-reactive WMI strain showed increased fear memory in a model of PTSD, the stress-enhanced fear learning behavior compared to the isogenic WLI strain^36^. Additionally, WMIs consume more alcohol than WLIs when tested using an operant licking procedure^36^. In human studies depression has been noted as a risk factor for dementia in females. Similarly, middle-aged WMI females show cognitive decline compared to middle-aged WLI females^37^. Together, these data establish WMI as a suitable model to study human depression. The WMI and WLI strains also differ in their brain and blood gene expression profiles^30^. A﻿ panel of blood transcriptomic markers, developed using the WMI strain, can diagnose major depression in humans. These blood transcriptomic markers are able to distinguish adolescent and adult subjects with major depression from those with no disorder with a high level of reliability^38–40^. Additionally, the expression of these markers correlated with depression symptoms in pregnant women^41^. These data provide tantalizing evidence that genetically determined gene expression differences between the WMI and WLI substrains can potentially lead to the discoveries of molecular mechanisms of depression in humans.

Full genome sequencing provides an abundance of genetic information (single nucleotide polymorphisms, inserts and deletions, and large structural variants) and can allow for comparative genomics between the rat model and humans. Comparing the genome of WMI and WLI to each other as well as the reference genome could provide insights to the underpinnings of their distinctive behavioral phenotypes. Because the WMI and WLI strains were both derived from WKY founders, we hypothesized that a small number of genetic variants between these strains contribute to behavioral and physiological differences in depression-associated traits between WMI and WLI. Here we describe the whole genome sequencing of these two strains using data obtained from three different platforms (Illumina xTen, Ion Proton, and 10X Chromium linked-read) and the identification of genetic variants between these strains.

## Results

To discover ﻿variants associated with the depression phenotype in WLI and WMI rats, whole genome sequencing data was obtained using three different platforms: Ion Torrent Proton, 10X Chromium and Illumina xTen from male WLI and WMI rats. Each technique covered an average depth of 41, 27 and 43 for both strains, respectively (Supplementary Fig. S1). X-chromosomal coverage was expected to be half of autosomal coverage but was found to be much higher on IonProton and Illumina X-ten sequencing results (Supplementary Fig. S1).

**Figure 1:**
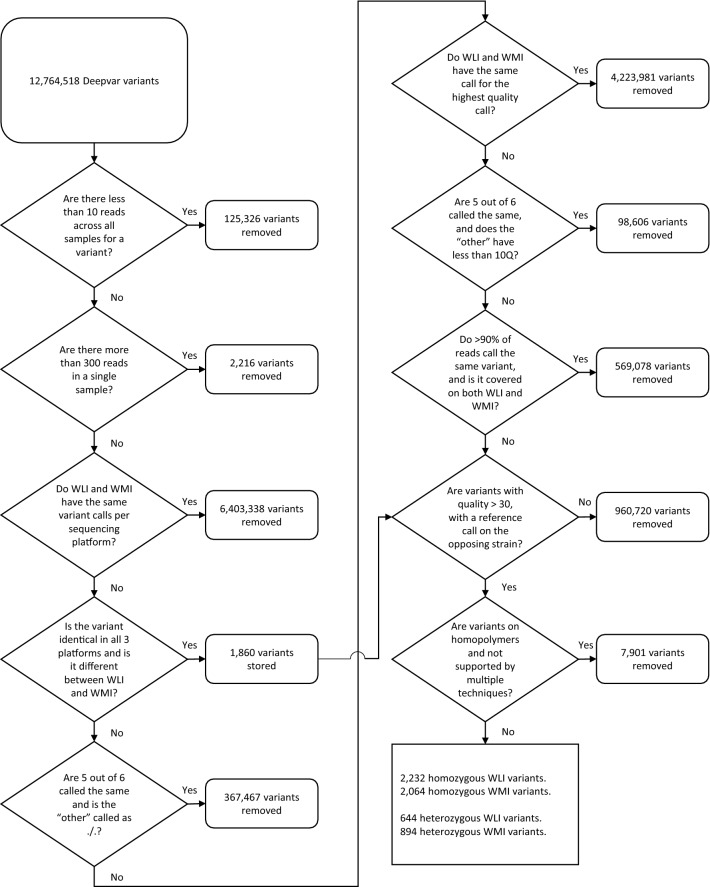
Flowchart of each filtering step and the number of variants removed per step. The initial 8 steps were performed in Python, the last 2 were performed in R.

Sequencing data were mapped to the rat reference genome rn6 using bwa^42^ (Illumina and IonProton data) or LongRanger (10X Chromium data). The resulting bam files were used as the input to DeepVariant^43^ to report genomic variants (i.e. SNPs and small indels) for each sample. GLNexus^44^ was then used to conduct a joint analysis of variants across all six samples. Over 12 million unique variants were identified before filtering. The analysis workflow was designed to take full advantage of the data provided from three sequencing technologies. We were interested in variants that have a Phred quality score above 30, have a clear call for either reference, homozygous or heterozygous, have no matching calls between WLI and WMI and must not have both a high quality reference and alternative allele called on different sequencing platforms within the same strain (Fig. 1).

**Figure 2:**
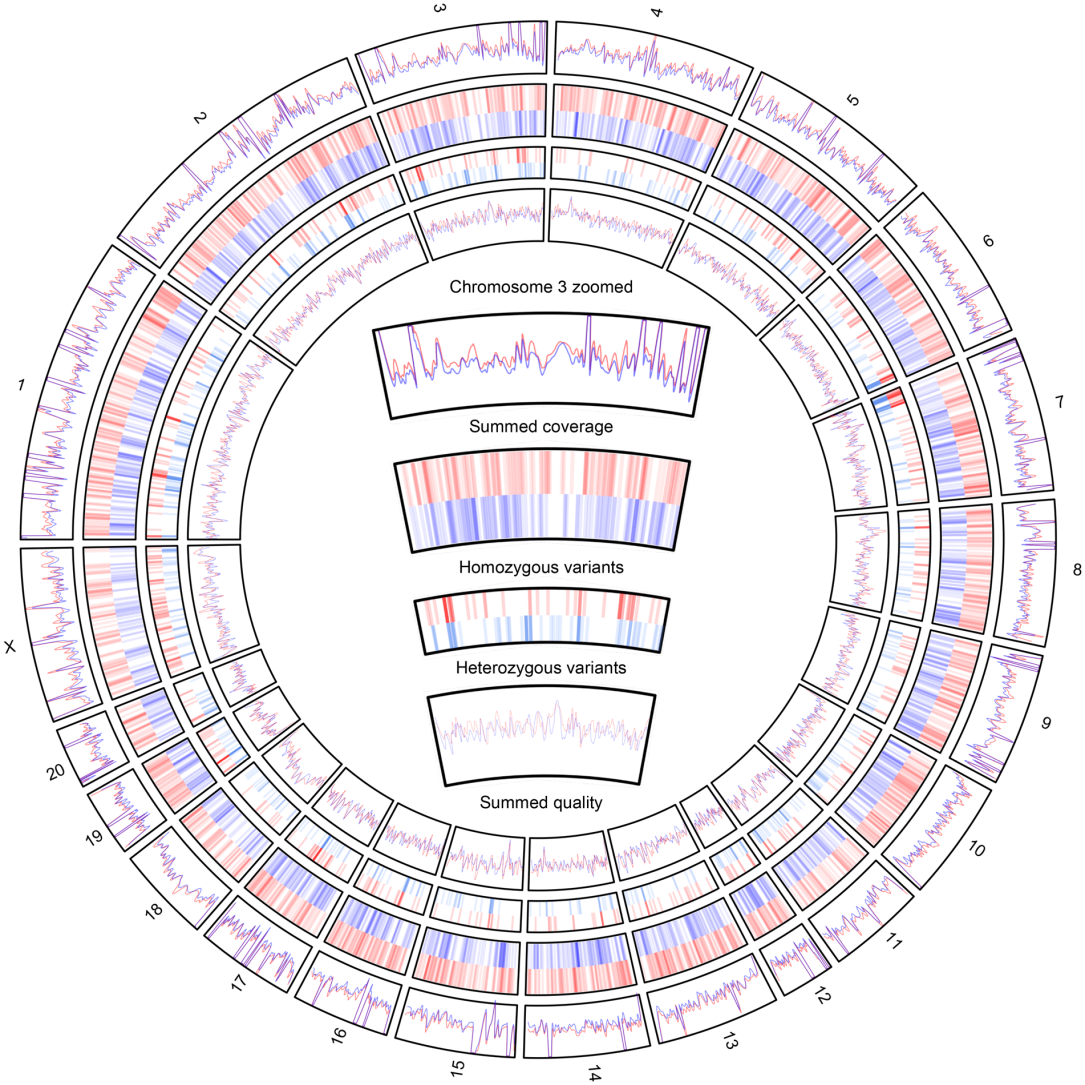
From outside to inside: (1) Smoothed summed coverage of variant calls per technique for WMI samples (blue) and WLI samples (red). (2) Hotspots of homozygous SNPs on each chromosome found only in WMI (Blue) or WLI (Red). (3) Hotspots of heterozygous variants on each chromosome found only in WMI (light blue) or WLI (light red). (4) Smoothed summed quality of variant calls per technique for WMI samples (blue) and WLI samples (red). * Chromosome Y excluded.

Phred quality scores and coverage varied greatly per genomic region (Fig. 2 track 1 and 4). A large portion of the variants had a Phred quality score below 10 and were excluded from subsequent analysis. In total, 99465, 25937, and 6454 homozygous variants had a Phred quality score greater than 10, 20, and 30 in at least one sample, respectively. For heterozygous calls the number of variants were ~ 3 million, ~ 1 million and ~ 200 thousand, for quality scores of 10, 20 and 30 respectively. The number of high-quality calls for homozygous variants varied per sequencing technology (Supplementary Fig. S2).

The majority of high confidence heterozygous calls came from a single technique, Ion proton (Supplementary Fig. S2). Closer inspection revealed that the majority (> 90%) of these calls was detected on homopolymeric nucleotide sequences. In addition, approximately 95% of these were deletions rather than SNPs, further confirming that these calls are due to errors in base calling homopolymeric sequences. To filter out this common sequencing error, all deletions on homopolymeric regions which were not supported by at least one other sequencing technique were removed (Supplementary Fig. S3).

As a final result, 2232 and 2064 homozygous high confidence variants were discovered on WLI and WMI respectively (Fig. 2 track 2). The majority were insertions (45.3%) followed by SNPs (36.9%), and finally deletions (17.8%) (Table 1). Of these SNPs, approximately 57% were transitions, meaning a purine nucleotide was mutated to another purine or a pyrimidine nucleotide to another pyrimidine. The other 43% were transversion SNPs, in which a purine was replaced by a pyrimidine or vice versa (Table 1). A total of 655 and 894 heterozygous variants were identified for WLI and WMI (Fig. 2 track 3). It should be noted that the heterozygous variants contained higher coverage than average as compared to homozygous variants (Supplementary Fig. S4). This implies a large portion of these could be homozygous SNPs aligned to collapsed regions on the reference genome.

**Table 1:** Overview of the number of variants, insertions and deletions in the final selection per strain.

	WLI	WMI
Transition SNP	478	428
Transversion SNP	325	356
Insertions	1090	855
Deletions	339	425

In addition, 79 and 119 homozygous variants were identified for WLI and WMI, respectively, with a Phred quality score of at least 10 in all three sequencing technologies. Though verified across technologies, quality scores cannot be simply summed. For certitude these were not included in the final selection.

We used SnpEff^45^ to identify the impact, location (Table 2), and the nearest gene in proximity of these variants. About half of the variants (52%) are located within intergenic regions, whilst some (a total of 62) variants fall within exons, 2432 are within introns, and 450 are located within 5 KB upstream of a gene (Supplementary Table S1).

**Table 2:** Position of selected variants in regions of interest.

Type (alphabetical order)	WLI	WMI
3 prime UTR variant	12	11
5 prime UTR variant	3	4
Downstream gene variant	177	117
Frameshift variant	6	5
Intergenic region	1440	1334
Intragenic variant	1	5
Intron variant	874	810
Missense variant	9	3
Non coding transcript exon variant	5	2
Splice acceptor variant	0	0
Splice donor variant	1	4
Splice region variant	7	7
Stop lost	1	0
Synonymous variant	5	0
Upstream gene variant	177	128
Total	2718	2430

In total, 1491 unique genes were in closest proximity to the final selection of homozygous variants across both strains. Of these, 744 genes were found in WMI and 866 in WLI (119 were found in both strains). These SNPs and indels are distributed across the entire genome and no genomic region shows enrichment of variants. However, three separate regions (1 kb) on WMI contained up to 5 SNPs on chromosomes 1,4 and 14. One of these clusters was positioned within the 3’ UTR of Zfp418. The other SNPs were located within intergenic regions.

In total, 9 WMI variants and 11 WLI variants were estimated to have a large impact on the final protein product. These included changes to splice sites, missense mutations, loss of stop codons or frameshifts (Table 3). These genes included *Asxl1*, *Zfp292*, *Wrap73*, *Col5a3*, *Abcc5*, *Fscn1*, *Wdfy3*, *Pou6f2*, *Svil*, *Prlr*, *Gnat2*, *Slc30a7*, *Kdm5a*, *Slco1a2*, *Nlrp1a*, *Crlf3*, *Tpcn1*, *Pigr*, *Pou6f2* and *Reep3*. Among these genes, *GNAT*2 has a variant in human (rs6537837) that was reported to be associated with unipolar depression with a genome wide significance of p = 1e−6^46^, while *Prlr* and *Nlrp1a* were implicated in depression-like behavior in animal models^47,48^. Further, *Pou6f2*, *Kdm5a*, *Reep3*, *Wdfy3* have been implicated in psychiatric diseases such as autism^49–51^ and *Pigr* was found to be involved in response to psychological stress^52,53^.

**Table 3:** Overview of variants of high and moderate impact, their impact and the gene affected.

Strain	Chr	Position	ALT	Gene name	Ensembl ID	Modification
WMI	3	148895880	TA	*Asxl1*	ENSRNOG00000001603	Splice donor variant & splice region variant & intron variant
WMI	5	50287827	A	*Zfp292*	ENSRNOG00000031031	Missense variant
WMI	5	171455130	C	*Wrap73*	ENSRNOG00000014805	Splice donor variant & splice region variant & intron variant
WMI	8	21788512	A	*Col5a3*	ENSRNOG00000020525	Missense variant & splice region variant
WMI	11	84399496	C	*Abcc5*	ENSRNOG00000029178	Frameshift variant
WMI	12	13660098	C	*Fscn1*	ENSRNOG00000056585	Frameshift variant
WMI	14	9266419	GA	*Wdfy3*	ENSRNOG00000061121	Frameshift variant
WMI	17	49440318	G	*Pou6f2*	ENSRNOG00000013237	Splice donor variant & intron variant
WMI	17	55289842	T	*Svil*	ENSRNOG00000018110	Missense variant
WLI	2	60302395	CCT	*Prlr*	ENSRNOG00000057557	Frameshift variant & splice region variant
WLI	2	210884044	A	*Gnat2*	ENSRNOG00000019296	Missense variant
WLI	2	218889177	C	*Slc30a7*	ENSRNOG00000013912	Stop lost
WLI	4	152938803	T	*Kdm5a*	ENSRNOG00000010591	Missense variant
WLI	4	176505968	T	*Slco1a2*	ENSRNOG00000031249	Splice donor variant & intron variant
WLI	10	57738003	A	*Nlrp1a*	ENSRNOG00000023143	Missense variant
WLI	10	67392591	CA	*Crlf3*	ENSRNOG00000050657	Frameshift variant & splice region variant
WLI	12	41544356	C	*Tpcn1*	ENSRNOG00000059344	Missense variant
WLI	13	47589399	G	*Pigr*	ENSRNOG00000004405	Missense variant
WLI	17	49440316	AG	*Pou6f2*	ENSRNOG00000013237	Frameshift variant
WLI	20	22913769	A	*Reep3*	ENSRNOG00000000645	Missense variant

We identified 75 and 70 SNPs on the X-chromosomes of WLI and WMI, respectively. SNPeff identified 8 as downstream variants, 46 as intergenic, 14 within introns, 5 upstream of genes and 2 in the 3’ UTR for WLI; 3 downstream variants, 51 within intergenic regions, 10 within introns, 4 upstream of genes, 1 in the 3’UTR, and 1 on a splice site/intron for WMI. Of these, two intron variants in WLI fell within HTR2C, a gene associated with depression^54,55^ schizophrenia^56,57^, and stress response^58,59^. One intron variant on WLI fell within Il1rapl1, a gene associated with autism^60,61^ and schizophrenia^62^. Additionally, an intron variant in WMI fell within CDKL5, a gene associated with neurodevelopmental disorders such as seizures and autistic-like symptoms^63,64^. No SNPs of high quality were identified on the Y-chromosome for either strain.

Previous research identified 101 genes that were significantly differentially expressed between WMI and WLI brain tissue^30,65^. We found 232 SNPs or indels located within or near these differentially expressed genes. Out of these variants, 128 fell in intergenic regions, 95 within intron variants, 4 upstream gene variants, 3 downstream gene variants and 2 within the 3'prime UTR of genes (Supplementary Table S2).

We also leveraged Gene Ontology-term (GO-term) and KEGG-term enrichment analysis using G-profiler^66^ to explore the biological functions of genes in close proximity to sequence variants. We found an over-representation of several neurogenesis, behavioral and locomotion related pathways. Over-represented terms for WLI included locomotion, behavior, nervous system development, neuron projection and neurogenesis (Supplementary Table S3). Over-represented terms for WMI included Par-3-KIF3A-PKC-zeta complex, actin-mediated cell contraction, neuronal related and cellular stress related pathways (Supplementary Table S3).

Of the genes found in close proximity to high impact variants (based on SNPeff annotations) in WLI, 30 were annotated with the GO-term neuron to neuron synapse (GO:0098984). We further examined these genes using GeneCup^67^, an online tool that allows us to conduct automated searches for genes associated with depression, addiction, stress, or other psychological afflictions from PubMed. We found 23 genes that were associated with psychiatric disease in previous research. These genes included; *Syt1*, *Stxbp5*, *Sorcs2*, *Rs1*, *Ptprd*, *Prkcz*, *Pdlim5*, *Lyn*, *Itga8*, *Igsf11*, *Grm3*, *Erbb4*, *Epha7*, *Epha4*, *Dlgap1*, *Dgki*, *Cdkl5*, *Cacna1c*, *Atp2b2*, *Ank2*, *Als2*, *Add3*, *Adcy8* (Supplementary Table S4).

Lastly, we validated our genome sequencing results by selecting 224 SNPs, half unique to each strain, and using multiplex PCR to amplify each target region (150 bp flanking each variant) in genomic DNA collected from 8 rats, including four WMI and four WLI with equal number of males and females. We then constructed sequencing libraries using these PCR products and sequenced them on an Illumina instrument. We were able to obtain PCR products from 89 and 87 primers sets targeting WLI and WMI specific variants, respectively. Among them, 75 WLI and 76 WMI targets met the following two criteria: 1. homozygous alternative in at least three rats of the target strain; 2. homozygous alternative in none of the rats of the opposite strain. Therefore, the positive rate of our stringent empirical validation using 8 rats was 85.8%.

## Discussion

The goal of this research is to give us genetic markers for WLI and WMI in context of other strains in reduced complexity crosses and to give us candidate variants for immediate scrutiny of linkage to depression. We used three leading next-generation sequencing technologies to obtain a combined coverage of approximately 100X for each genome of two closely related inbred rat strains, the WMI and WLI. We identified 4296 homozygous variants with high fidelity that are located in close proximity to 1491 unique genes that differ between these two strains. The SNPs and indels identified in this dataset offer new opportunities for the identification of genes related to the phenotypic differences between the WLI and WMI strains.

The WMI strain was originally characterized as a genetic model of depression^29–31^. However, since their development based on behavior in the forced swim test, it has been suggested that this task measures coping style^68^, thus WLIs and WMIs may be a genetic model of stress coping style differences. Still, their blood RNA-seq results contributed to the identification of a blood-based transcriptomic panel for human depression^38,39,69^. Furthermore, the strains differ in behavioral and hormonal responsiveness to acute and chronic stress^33,36^, and in drug-taking behaviors^36,70^. Thus, regardless whether the WMI is a genetic model of depression-like behavior or passive coping, the fact that many WMI phenotypes show behavioral parallels with human stress-related disorders, including major depression, inform its significance.

Each of the three sequencing methods we used has its own merits and flaws. For example, compared to the widely used Illumina platform, the Ion Torrent platform provides high quality data at a lower cost. However, it suffers at homopolymer regions. The 10 × Chromium linked reads technology attaches barcodes to high molecular weight DNA before library preparation and can detect large structural variants. But obtaining good quality HMW DNA is technically challenging and is associated with increased cost. Further, when utilizing sequencing data from a single technique, technical biases are likely to make their way into the final result. By removing variants called differently by sequencing platforms, the technical bias is mitigated across the final selection of variants.

We used DeepVariant to identify SNP and small indels across all sequencing techniques^43^. Deepvariant has been shown to outperform GATK in different tests, especially in calling indels^71^. It also handles data from diverse sequencing platforms without additional calibration. We used LongRanger to map the 10X linked reads sequencing data to the reference genome because it incorporates the molecular barcodes into the mapping algorithm. Following DeepVariant analysis, we used GLNexus to conduct a joint analysis to obtain a list of raw genomic variants. Joint analysis empowers variant discovery by leveraging population-wide information from a cohort of multiple samples, allowing us to detect variants with great sensitivity and genotype samples as accurately as possible^72^.

With the combination of different sequencing methods, a higher certainty of variant calling between WLI and WMI has been made possible, though throughout this experiment strict filtering was performed. We first removed any variants detected with any certainty in both WLI and WMI, because we are interested in the differences between these two strains, rather than their common differences to the reference genome. One caveat of this approach is that variants incorrectly called by DeepVariant (e.g. due to low coverage in a single method) can lead to the exclusion of potentially interesting targets. Similarly, a strict quality score cutoff of 30 was used whilst un-opposed by any other sample with a Phred quality score of 10 or higher. Based on the abundance of data we have collected, including thousands of identified variants, we decided that 1 in 1000 variants was a sensible cutoff to avoid hundreds of false positives in the final set of variants.

The current reference genome (rn6) consists of 75697 contigs and 1395 scaffolds with N50 lengths of 100.5 KB and 14.99 Mb respectively. These sequences combine into a golden path of approximately 2.8 billion bases. Due to the fragmented nature of the reference genome, the identification of structural variants has proven to be difficult. One example of this is that it is often not possible to establish whether sequence variation is strain specific or related to a problem with the reference genome. In addition to the 4,296 high quality homozygous variants discovered in this research, an additional 15268 variants were discovered in either WLI or WMI with no significant coverage or significant phred score on the opposing strain (called as ./. ). Without a high quality read on both strains we cannot verify newly discovered variants. These low quality calls could be caused by heterozygosity, low coverage or overlapping variants.

We were able to confirm 151 out of 176 (85.8%) of the variants in 8 additional samples using an independent method. We found 232 SNPs in proximity of 101 genes which were significantly differentially expressed in previous research^30,65^. A large portion of these fell within intergenic regions that are unlikely to affect gene expression (Supplementary Table S2). Others, like those upstream of the TSS, downstream of genes r within introns are more likely to regulate expression levels. Together, these results provided high confidence on the accuracy of our analysis results.

Previous research has shown several sex specific differences between the WLI and WMI strains. Although depression-like behavior is different between these strains in both adults and adolescents in males, strain difference is only found in adult females and not in adolescent females. In addition, in measures of anxiety behavior, strain difference is found in adult males but not in adult females^31^. In this investigation several SNPs were identified on chromosome X. A few of these variants are located in the intron of genes previously associated with psychiatric conditions. However, this does not guarantee these variants are directly responsible for the phenotypic differences between the WMI and WLI.

Cross comparison of the genomic positions of variants discovered on WLI and WMI with variants discovered in a panel of 44 inbred rat strains (unpublished data). Out of the 2232 variants on WLI and the 2064 variants on WMI, 1215 and 856 were unique to each strain, respectively. This indicates these variants are likely denovo to WMI and WLI. Future research can verify the origin of the variants identified in this research.

In this investigation 655 and 894 heterozygous variants were discovered on WLI and WMI respectively. Despite both strains being fully inbred, there is a chance that de-novo mutations could propagate as heterozygous variants within each substrain. A look at the coverage of these positions reveals an average two-fold coverage, implying these variants are homozygous on collapsed regions on the reference genome, or duplicated and mutated within either strain. With an updated reference genome these regions could be resolved and can contribute in a meaningful way to the identification of variants that contribute to phenotypic differences between WLI and WMI substrains.

The current reference genome (Rnor_6) is likely to contain many errors^73^, this means some caution is required when identifying variants for WLI or WMI strains based on comparison to the current rat reference genome. There is a chance that variants found in both strains could potentially be due to base level errors in the reference, i.e., there is no variant present at all. Similarly, when a variant is only reported in strain A, there exists a small chance that the variant actually is located on strain B (i.e. the base level error in the reference happens to be the same as the sequence in strain B). Thus, a small percentage of the reported mutations in WLI strain could potentially be present in WMI. This might contribute, to some degree, to the enrichment of neuronal GO-term annotation for genes located within the vicinity of WLI sequence variants.

GO-term annotation enrichment for genes in the nearest proximity of variants detected in WLI included locomotor behavior and neuron projection. This provides some evidence that these variants could be capable of producing an impact on behavior, however this will require further investigation. As locomotory behavior is a complex trait, a combination of variants can be causal. For WMI the terms: neuron to neuron synapse (GO:0098984), nervous system development (GO:0007399), generation of neurons (GO:0048699), and finally, the Par-3-KIF3A-PKC-zeta complex (CORUM:899) was significantly over-represented. The Par-3-KIF3A-PKC-zeta complex is interesting as both parts are in proximity of variants detected on WMI and it is involved in the establishment of neuronal polarity^74^.

The ancestral WKY strain was noted for its highly variable behavior between individuals^21,22^. With the discovery of variants associated with psychiatric phenotypes in both strains it should be kept in mind that variants could have been both selected for and against. In addition, as discussed above, there is a small chance some variants are located on the opposite strain due to potential errors in the reference genome. For this reason, we have only included variants which are different between WLI and WMI and not those that are different relative to the reference genome.

Lastly, our current analysis is focused on SNPs and indels. Additional data are required to accurately identify other genomic differences, such as large insertions, deletions, tandem repeats, etc. Further, finding genetic differences between the WMI and WLI strains is only the first step in identifying causal variants for many of the phenotypes that are different between them. These causal variants can be identified by genetic mapping using F2 offspring from WMI and WLI parents. This strategy of generating a reduced complexity cross to map causal genetic variants has many successful precedents^10–12^ and could lead to identification of novel genes and variants causing phenotypic variation between these two strains (e.g., depression-like behavior, drug abuse, memory, aging, stress responsiveness).

## Methods

### Animals

Liver tissue from 4 adult WLI (2 males and 2 females) and 4 adult WMI (2 males and 2 females) rats were collected. Rats used for genome sequencing were randomly selected from the colony. Blinding was not relevant in this study because rats were not subjected to any experimental conditions. Equal amounts of tissue from males and females were pooled for each strain (total weight = 20 mg). DNA were extracted using the Qiagen DNeasy blood and tissue kit (Cat# 69506).

### Whole genome sequencing

For sequencing using the HiSeq X Ten instrument, DNA whole genome shotgun sequencing libraries were generated using 200 ng of genomic DNA as input for the TruSeq Nano DNA Library Prep Kit (Illumina). Indexed libraries were sequenced as pools of eight samples on a full slide (8 lanes) on an Illumina HiSeq X Ten sequencer using HiSeq X Ten v2.5 reagents. For sequencing using the Ion Torrent instrument, 1 μg of genomic DNA was sheared to an average size of 200 bp using a Covaris S2 Sonicator. Then 500 ng of the sheared DNA was used to prepare libraries for sequencing using the AB Library Builder™ Fragment library Kit on a Library Builder system. Libraries were used without amplification and size selected on a 2% Pippin Prep gel. After quantification using qPCR, the libraries (190 pg) were then used to prepare beads for sequencing using an Ion Torrent One Touch instrument. DNA on these beads then sequenced on an Ion Torrent Proton sequencer using Hi-Q chemistry and a P1 chip. For 10X Chromium sequencing, the Qiagen MagAttract HMW DNA kit was used for DNA isolation. Sequencing library was then constructed from 1 ng of high molecular weight (~ 50 kb) genomic DNA using the Chromium Genome Library kit and sequenced on Illumina Hi-Seq (150 bp PE).

### Mapping

Illumina and Ion proton data were mapped to the rat reference genome (rn6) using bwa (reference). 10 × Chromium data were mapped to rn6 using LongRanger (ver 2.2.2). DeepVariant (ver 1.0.0) was used to call SNPs and small indels from the bam files and GLnexus was used for joint calling of variants.

### Analysis

Variant identification was performed separately for each strain and sequencing method. A total of 6 samples spread over 2 strains and 3 sequencing technologies were analyzed. Variants with less than 10 reads across all samples or more than 300 on a single sample for a variant were removed. Variants with the same highest quality call for WLI and WMI were removed. Variants with an identical call for all three sequencing technologies within either WLI or WMI were stored for further analysis. Variants with 5 out of 6 uncertain calls (./.) were removed. Variants with the same highest quality call for WMI and WLI were removed. Variants with 5 out of 6 identical calls of which the last had a quality score less than 10 were removed. If the majority (> 90%) of reads were of the same variant call across all reads and both strains shared at least 25% of all reads, the variant was removed.

Variants were selected based on the highest quality call per method and removed if disputed by variants called on another sequencing method with call quality of at least 30 within the same strain. Only variants were included in which the call for WLI differed from WMI and one of two strains was called as 0/0 (reference allele). Finally, all deletions on a position consisting of two identical nucleotides (homopolymeric) which were not supported by multiple sequencing techniques were removed (Fig. 1). The final selection was exported to VCF per strain and type of call (homozygous or heterozygous). Figure 2 was generated with the aid of the Circos R package^76^.

SnpEff (v4_3t_core) was used for nearest gene identification, impact estimation and annotation of the VCF for selected variants^45^. Impact and nearest genes were estimated separately per strain, as well as heterozygous and homozygous variants. Variants marked as high or moderate impact were separated and placed in Table 3. The annotated VCF is available for reference. g:Profiler version e101_eg48_p14_baf17f0 was used for GO-term enrichment analysis, standard settings were used, no background dataset was utilized^66^. RatsPub^67^) was used to explore a small set of genes nearest to variants enriched with the GO-term: neuron to neuron synapse (GO:0098984).

### Validation of variants and small indels by targeted re-sequencing

Ear punches from four WLI, four WMI (equal number of males and females) were used to extract genomic DNA. A total of 112 variants unique to WMI and 112 variants unique to WLI were selected from the final list of variants, with approximately equal distribution across the genome. Individual primer pairs were designed using Batch Primer 3 (http://probes.pw.usda.gov/batchprimer3/) at default settings for generic primers with total amplicon size set as an optimum of 100 bp with the amplified region containing the target SNP (or region of interest). The primer sequences and genomic DNA were submitted to Floodlight Genomics (FG, Knoxville, TN) for processing using a Hi-Plex targeted sequencing approach^75^. The Hi-Plex approach pools primers to PCR amplify targets and adds a barcode sequence during the amplification process. The resulting target library is then sequenced on an Illumina instrument. Data were then aligned to the fasta file containing the targeting target variants using bwa. Genotypes for each sample were called using Deepvariant.

### Ethics approval and consent to participate

All procedures were approved by the Animal Care and Use Committee of The University of Tennessee Health Science Center and were conducted in accordance with the NIH Guidelines concerning the Care and Use of Laboratory Animals. All methods are reported in accordance with ARRIVE guidelines.

## Supplementary Information

Supplementary Table S1.

Supplementary Table S2.

Supplementary Table S3.

Supplementary Table S4.

Supplementary Legends.

Supplementary Figure 1.

Supplementary Figure 2.

Supplementary Figure 3.

Supplementary Figure 4.

## Data Availability

The datasets used and/or analyzed during the current study are available from the corresponding author on reasonable request. Analysis scripts and annotated vcf files are available from github (https://github.com/tristandejong/WLI_WMI_analysis).

## References

[1] WHO (2018). Disease Burden and Mortality Estimates.

[2] Sullivan PF, Neale MC, Kendler KS (2000). Genetic epidemiology of major depression: Review and meta-analysis. Am. J. Psychiatry.

[3] Fernandez-Pujals AM (2015). Epidemiology and heritability of major depressive disorder, stratified by age of onset, sex, and illness course in generation Scotland: Scottish Family Health Study (GS:SFHS). PLoS ONE.

[4] Wang K, Gaitsch H, Poon H, Cox NJ, Rzhetsky A (2017). Classification of common human diseases derived from shared genetic and environmental determinants. Nat. Genet..

[5] Flint J, Kendler KS (2014). The genetics of major depression. Neuron.

[6] CONVERGE Consortium (2015). Sparse whole-genome sequencing identifies two loci for major depressive disorder. Nature.

[7] Hyde CL (2016). Identification of 15 genetic loci associated with risk of major depression in individuals of European descent. Nat. Genet..

[8] Wray NR (2018). Genome-wide association analyses identify 44 risk variants and refine the genetic architecture of major depression. Nat. Genet..

[9] Howard DM (2019). Genome-wide meta-analysis of depression identifies 102 independent variants and highlights the importance of the prefrontal brain regions. Nat. Neurosci..

[10] Bryant CD (2020). Facilitating complex trait analysis via reduced complexity crosses. Trends Genet..

[11] Kumar V (2013). C57BL/6N mutation in cytoplasmic FMRP interacting protein 2 regulates cocaine response. Science.

[12] Mulligan MK (2019). Identification of a functional non-coding variant in the GABA A receptor α2 Subunit of the C57BL/6J mouse reference genome: Major implications for neuroscience research. Front. Genet..

[13] Louis WJ, Howes LG (1990). Genealogy of the spontaneously hypertensive rat and Wistar-Kyoto rat strains: Implications for studies of inherited hypertension. J. Cardiovasc. Pharmacol..

[14] Kurtz TW, Montano M, Chan L, Kabra P (1989). Molecular evidence of genetic heterogeneity in Wistar-Kyoto rats: Implications for research with the spontaneously hypertensive rat. Hypertension.

[15] Paré WP, Redei E (1993). Sex differences and stress response of WKY rats. Physiol. Behav..

[16] Solberg LC (2004). Sex- and lineage-specific inheritance of depression-like behavior in the rat. Mamm. Genome.

[17] Malkesman O (2006). Two different putative genetic animal models of childhood depression. Biol. Psychiatry.

[18] Tizabi Y (2010). Effects of nicotine on depressive-like behavior and hippocampal volume of female WKY rats. Prog. Neuropsychopharmacol. Biol. Psychiatry.

[19] De La Garza R, Mahoney JJ (2004). A distinct neurochemical profile in WKY rats at baseline and in response to acute stress: Implications for animal models of anxiety and depression. Brain Res..

[20] Vinod KY (2012). Dysfunction in fatty acid amide hydrolase is associated with depressive-like behavior in Wistar Kyoto rats. PLoS ONE.

[21] Dugovic C, Solberg LC, Redei E, Van Reeth O, Turek FW (2000). Sleep in the Wistar-Kyoto rat, a putative genetic animal model for depression. NeuroReport.

[22] Baum AE (2006). Test- and behavior-specific genetic factors affect WKY hypoactivity in tests of emotionality. Behav. Brain Res..

[23] Solberg LC, Olson SL, Turek FW, Redei E (2001). Altered hormone levels and circadian rhythm of activity in the WKY rat, a putative animal model of depression. Am. J. Physiol. Regul. Integr. Comp. Physiol..

[24] Schaffer DJ, Tunc-Ozcan E, Shukla PK, Volenec A, Redei EE (2010). Nuclear orphan receptor Nor-1 contributes to depressive behavior in the Wistar-Kyoto rat model of depression. Brain Res..

[25] Hurley LL (2013). Antidepressant-like effects of curcumin in WKY rat model of depression is associated with an increase in hippocampal BDNF. Behav. Brain Res..

[26] Shoval G (2016). Prohedonic effect of cannabidiol in a rat model of depression. Neuropsychobiology.

[27] Kurtz TW, Morris RC (1987). Biological variability in Wistar-Kyoto rats. Implications for research with the spontaneously hypertensive rat. Hypertension.

[28] Paré WP, Kluczynski J (1997). Differences in the stress response of Wistar-Kyoto (WKY) rats from different vendors. Physiol. Behav..

[29] Will CC, Aird F, Redei EE (2003). Selectively bred Wistar-Kyoto rats: An animal model of depression and hyper-responsiveness to antidepressants. Mol. Psychiatry.

[30] Andrus BM (2012). Gene expression patterns in the hippocampus and amygdala of endogenous depression and chronic stress models. Mol. Psychiatry.

[31] Mehta NS, Wang L, Redei EE (2013). Sex differences in depressive, anxious behaviors and hippocampal transcript levels in a genetic rat model. Genes Brain Behav..

[32] Luo W (2020). Hypothalamic gene expression and postpartum behavior in a genetic rat model of depression. Front. Behav. Neurosci..

[33] Mehta-Raghavan NS, Wert SL, Morley C, Graf EN, Redei EE (2016). Nature and nurture: Environmental influences on a genetic rat model of depression. Transl. Psychiatry.

[34] Williams KA, Mehta NS, Redei EE, Wang L, Procissi D (2014). Aberrant resting-state functional connectivity in a genetic rat model of depression. Psychiatry Res..

[35] Mulders PC, van Eijndhoven PF, Schene AH, Beckmann CF, Tendolkar I (2015). Resting-state functional connectivity in major depressive disorder: A review. Neurosci. Biobehav. Rev..

[36] Lim PH (2018). Genetic model to study the co-morbid phenotypes of increased alcohol intake and prior stress-induced enhanced fear memory. Front. Genet..

[37] Lim PH (2018). Premature hippocampus-dependent memory decline in middle-aged females of a genetic rat model of depression. Behav. Brain Res..

[38] Pajer K (2012). Discovery of blood transcriptomic markers for depression in animal models and pilot validation in subjects with early-onset major depression. Transl. Psychiatry.

[39] Redei EE (2014). Blood transcriptomic biomarkers in adult primary care patients with major depressive disorder undergoing cognitive behavioral therapy. Transl. Psychiatry.

[40] Yu JS, Xue AY, Redei EE, Bagheri N (2016). A support vector machine model provides an accurate transcript-level-based diagnostic for major depressive disorder. Transl. Psychiatry.

[41] Redei EE, Ciolino JD, Wert SL, Yang A, Kim S, Clark C, Zumpf KB, Wisner KL (2020). Pilot validation of blood-based biomarkers during pregnancy and postpartum in women with prior or current depression. Transl. Psychiatry.

[42] Li H, Durbin R (2010). Fast and accurate long-read alignment with Burrows-Wheeler transform. Bioinformatics.

[43] Poplin R (2018). A universal SNP and small-indel variant caller using deep neural networks. Nat. Biotechnol..

[44] Yun T (2020). Accurate, scalable cohort variant calls using DeepVariant and GLnexus. Cold Spring Harbor Lab..

[45] Cingolani P (2012). A program for annotating and predicting the effects of single nucleotide polymorphisms, SnpEff: SNPs in the genome of Drosophila melanogaster strain w1118; iso-2; iso-3. Fly.

[46] Shi J (2011). Genome-wide association study of recurrent early-onset major depressive disorder. Mol. Psychiatry.

[47] Tian R-H, Bai Y, Li J-Y, Guo K-M (2019). Reducing PRLR expression and JAK2 activity results in an increase in BDNF expression and inhibits the apoptosis of CA3 hippocampal neurons in a chronic mild stress model of depression. Brain Res..

[48] Song A-Q (2020). NLRP1 inflammasome contributes to chronic stress-induced depressive-like behaviors in mice. J. Neuroinflamm..

[49] Napoli E (2018). Beyond autophagy: A novel role for autism-linked Wdfy3 in brain mitophagy. Sci. Rep..

[50] Chen K (2019). Drosophila histone demethylase KDM5 regulates social behavior through immune control and gut microbiota maintenance. Cell Host Microbe.

[51] Castermans D (2007). Identification and characterization of the TRIP8 and REEP3 genes on chromosome 10q21.3 as novel candidate genes for autism. Eur. J. Hum. Genet..

[52] Campos-Rodríguez R (2013). Stress modulates intestinal secretory immunoglobulin A. Front. Integr. Neurosci..

[53] Zallocco L (2021). Salivary proteome changes in response to acute psychological stress due to an oral exam simulation in university students: Effect of an olfactory stimulus. Int. J. Mol. Sci..

[54] Levchenko A (2020). NRG1, PIP4K2A, and HTR2C as potential candidate biomarker genes for several clinical subphenotypes of depression and bipolar disorder. Front. Genet..

[55] Hill SY, Jones BL, Haas GL (2020). Suicidal ideation and aggression in childhood, genetic variation and young adult depression. J. Affect. Disord..

[56] Zhang J-P (2016). Pharmacogenetic associations of antipsychotic drug-related weight gain: A systematic review and meta-analysis. Schizophr. Bull..

[57] Li J, Hashimoto H, Meltzer HY (2019). Association of Serotonin2c receptor polymorphisms with antipsychotic drug response in schizophrenia. Front. Psychiatry.

[58] Way BM, Brown KW, Quaglia J, McCain N, Taylor SE (2016). Nonsynonymous HTR2C polymorphism predicts cortisol response to psychosocial stress II: Evidence from two samples. Psychoneuroendocrinology.

[59] Avery BM, Vrshek-Schallhorn S (2016). Nonsynonymous HTR2C polymorphism predicts cortisol response to psychosocial stress I: Effects in males and females. Psychoneuroendocrinology.

[60] Bhat SS (2008). Disruption of the IL1RAPL1 gene associated with a pericentromeric inversion of the X chromosome in a patient with mental retardation and autism. Clin. Genet..

[61] Montani C (2017). The X-linked intellectual disability protein IL1RAPL1 regulates dendrite complexity. J. Neurosci..

[62] Lam M (2019). Comparative genetic architectures of schizophrenia in East Asian and European populations. Nat. Genet..

[63] Pizzo R, Lamarca A, Sassoè-Pognetto M, Giustetto M (2020). Structural bases of atypical whisker responses in a mouse model of CDKL5 deficiency disorder. Neuroscience.

[64] Weaving LS (2004). Mutations of CDKL5 cause a severe neurodevelopmental disorder with infantile spasms and mental retardation. Am. J. Hum. Genet..

[65] Raghavan NS (2017). Prepubertal ovariectomy exaggerates adult affective behaviors and alters the hippocampal transcriptome in a genetic rat model of depression. Front. Endocrinol..

[66] Raudvere U (2019). g:Profiler: A web server for functional enrichment analysis and conversions of gene lists (2019 update). Nucleic Acids Res..

[67] Gunturkun MH (2021). GeneCup: mine PubMed for gene relationships using custom ontology and deep learning. Cold Spring Harbor Lab..

[68] Molendijk ML, de Kloet ER (2019). Coping with the forced swim stressor: Current state-of-the-art. Behav. Brain Res..

[69] Redei EE (2021). Pilot validation of blood-based biomarkers during pregnancy and postpartum in women with prior or current depression. Transl. Psychiatry.

[70] Kim, P. *et al.* Rat reduced complexity model of oxycodone self-administration and stress responsiveness. Virtual NIDA Genetics and Epigenetics Consortium Meeting (2021).

[71] Supernat A, Vidarsson OV, Steen VM, Stokowy T (2018). Comparison of three variant callers for human whole genome sequencing. Sci. Rep..

[72] Brouard J-S, Schenkel F, Marete A, Bissonnette N (2019). The GATK joint genotyping workflow is appropriate for calling variants in RNA-seq experiments. J. Anim. Sci. Biotechnol..

[73] Ramdas S (2019). Extended regions of suspected mis-assembly in the rat reference genome. Sci. Data.

[74] Nishimura T (2004). Role of the PAR-3-KIF3 complex in the establishment of neuronal polarity. Nat. Cell Biol..

[75] Nguyen-Dumont T, Pope BJ, Hammet F, Southey MC, Park DJ (2013). A high-plex PCR approach for massively parallel sequencing. Biotechniques.

[76] Krzywinski M (2009). Circos: An information aesthetic for comparative genomics. Genome Res..

