# Supplementary material for: Whole genome sequencing of nearly isogenic WMI and WLI inbred rats identifies genes potentially involved in depression and stress reactivity
Source: Sci Rep. 2021 Jul 20;11:14774. doi: 10.1038/s41598-021-92993-4 (PMC8292482; doi:10.1038/s41598-021-92993-4)
Supplement: Supplementary file 5 — Supplementary Legends. [file 41598_2021_92993_MOESM5_ESM.docx]

**Supplementary figure 1.** Mean depth of coverage per chromosome per sample. Deepvariant called a total of 12,764,518 unique variants across 20 chromosomes plus X and Y with varying quality scores on either WLI or WMI samples. Depth of coverage is shown per A) WLI, IonProton, B) WLI 10X Chromium, C) WLI Illumina xTen, D) WMI IonProton, E) WMI 10X Chromium, F) WMI Illumina xTen.

**Supplementary figure 2.** A) Number of ALT calls by deepVariant. Samples are separated based on quality score: quality 10 = (p < 0.1), quality 20 = (p < 0.01), quality 30 = (p < 0.001). B) Number of HET calls by deepVariant in different samples separated by quality.

**Supplementary figure 3.** Total number of homozygous (ALT) and heterozygous (HET) variants after final selection before and after homopolymer removal per strain.

**Supplementary figure 4.** Coverage of reference called variants to alternative on opposing strains (REF), homozygous variants (ALT) and heterozygous variants per sequencing technology. The coverage of heterozygous variants is overal twice as high as reference calls to homozygous variants on the opposing strain and homozygous variants.

**Supplementary table 1.** Positions of variants relative to genes on the genome for both WLI and WMI. Some regions overlap in classification.

**Supplementary table 2.** Genes significantly differentially expressed in previous studies in proximity of SNPs.

**Supplementary table 3.** GO-term enrichment analysis for WLI and WMI.

**Supplementary table 4.** Overview of 23 out of 30 genes associated with the enriched GO-term: neuron to neuron synapse (GO:0098984) that have been associated with psychiatric disease in previous studies.
