# Supplementary figures and images for: Whole genome sequencing of nearly isogenic WMI and WLI inbred rats identifies genes potentially involved in depression and stress reactivity
Source: Sci Rep. 2021 Jul 20;11:14774. doi: 10.1038/s41598-021-92993-4 (PMC8292482; doi:10.1038/s41598-021-92993-4)

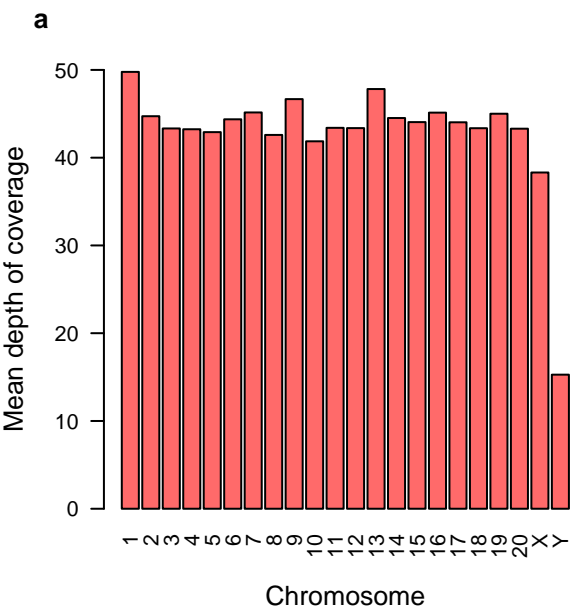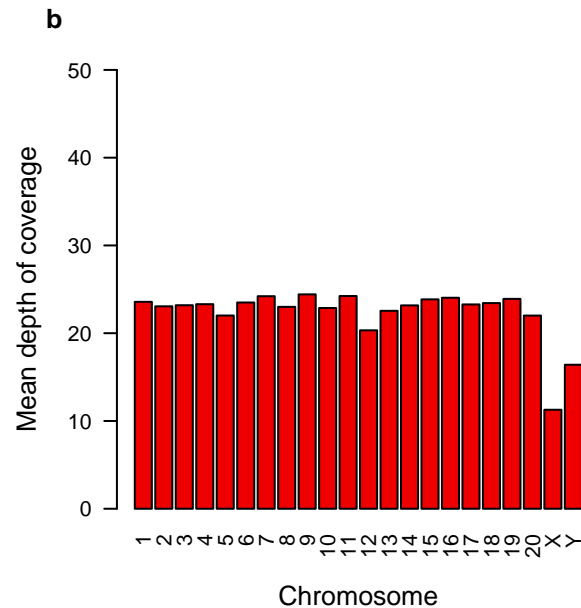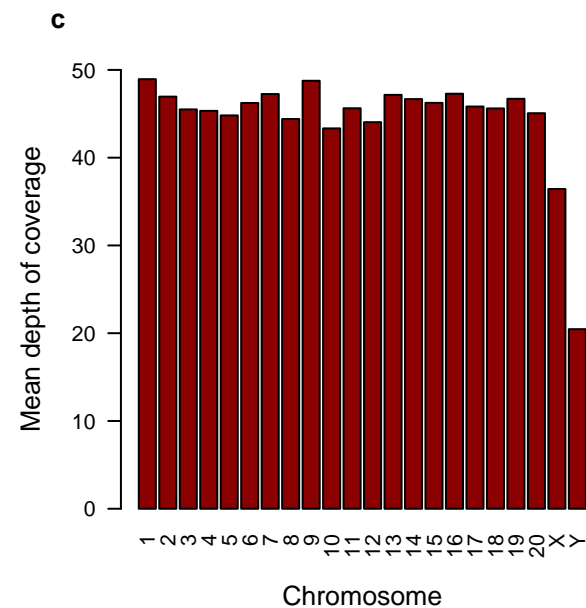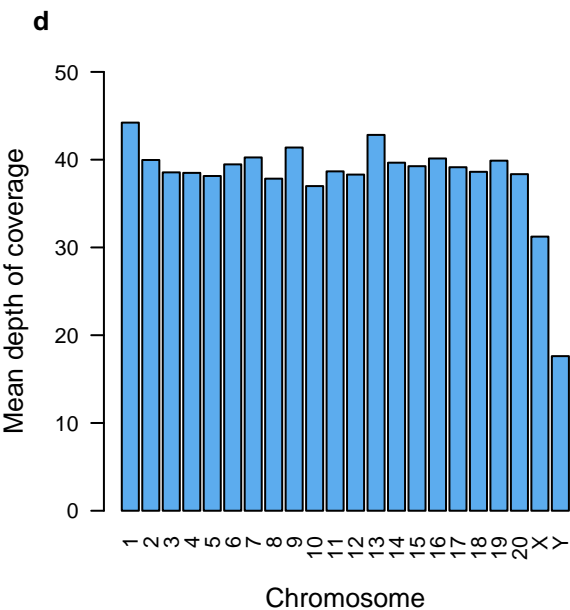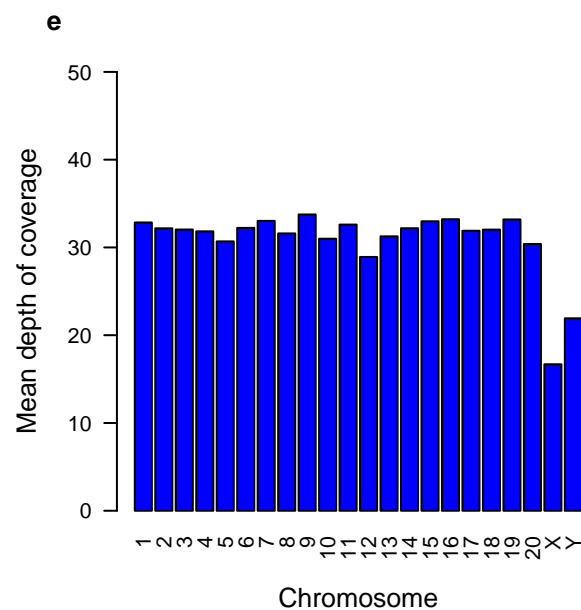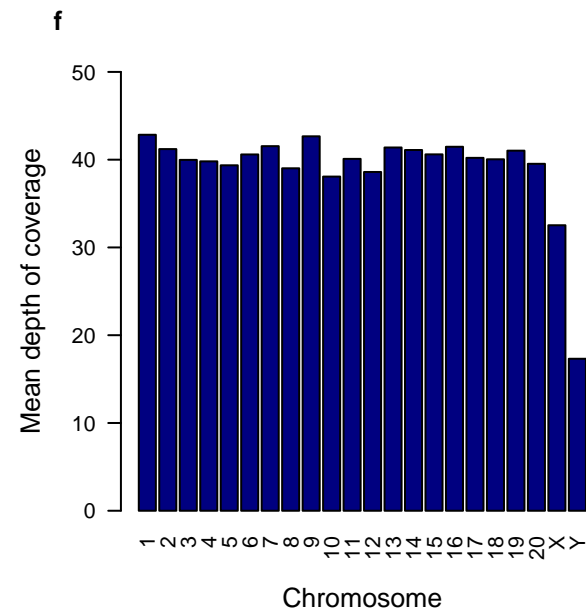

Supplement: Supplementary file 6 — Supplementary Figure 1. [file 41598_2021_92993_MOESM6_ESM.pdf]

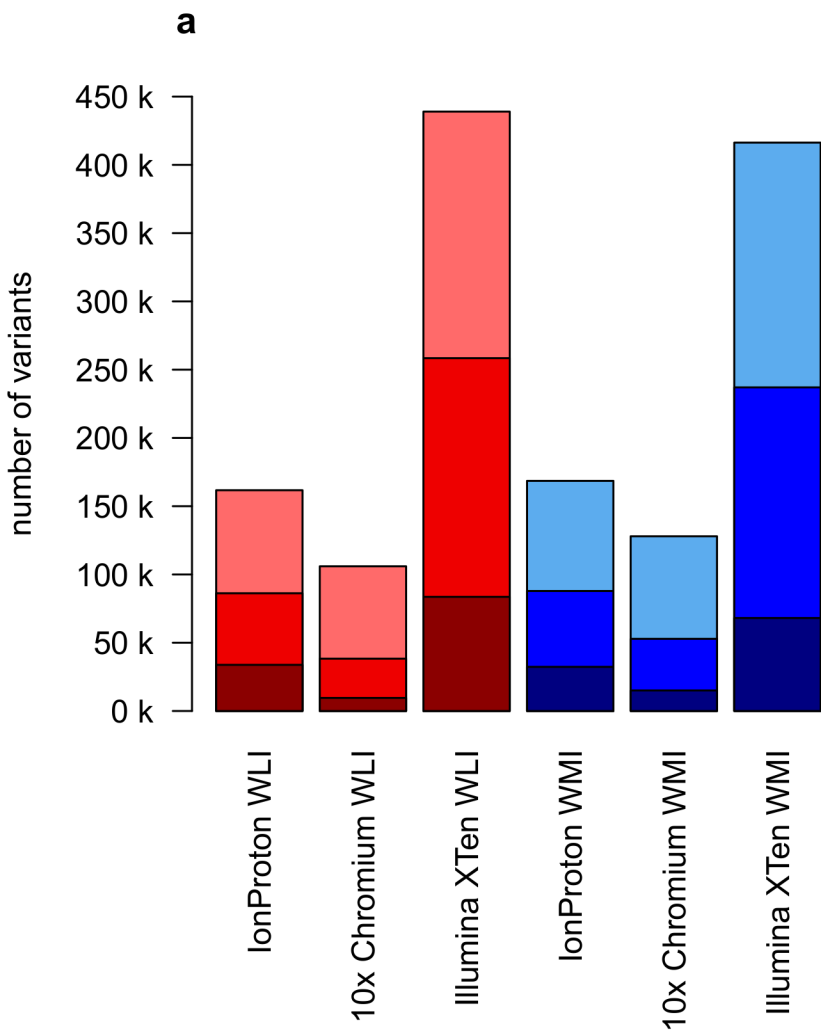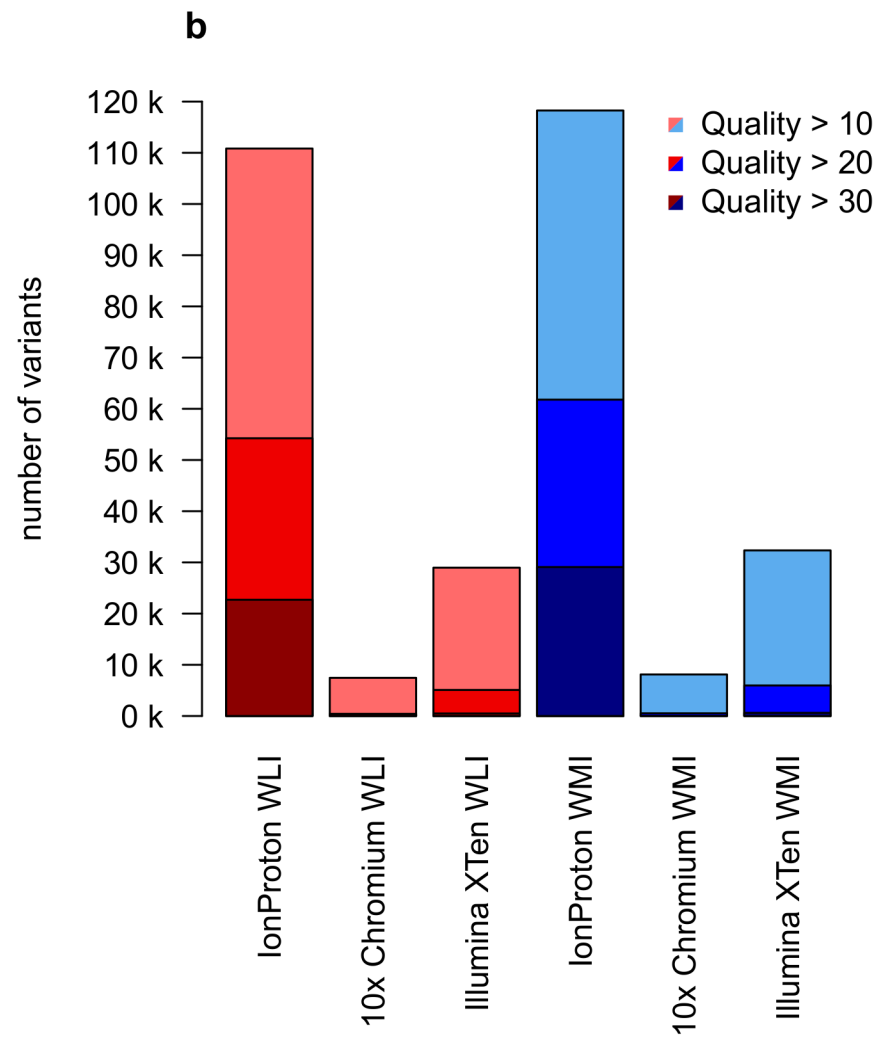

Supplement: Supplementary file 7 — Supplementary Figure 2. [file 41598_2021_92993_MOESM7_ESM.pdf]

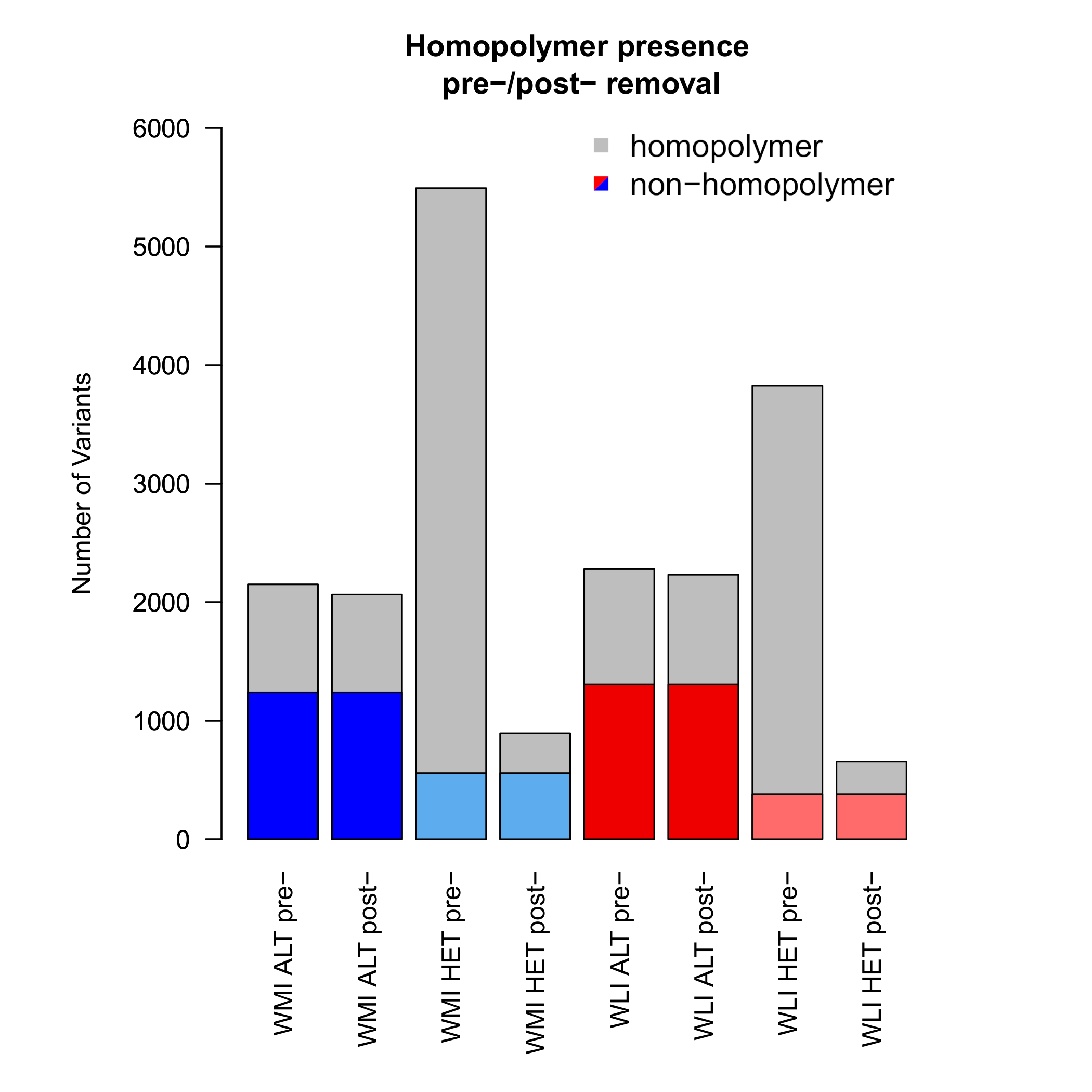

Supplement: Supplementary file 8 — Supplementary Figure 3. [file 41598_2021_92993_MOESM8_ESM.jpg]

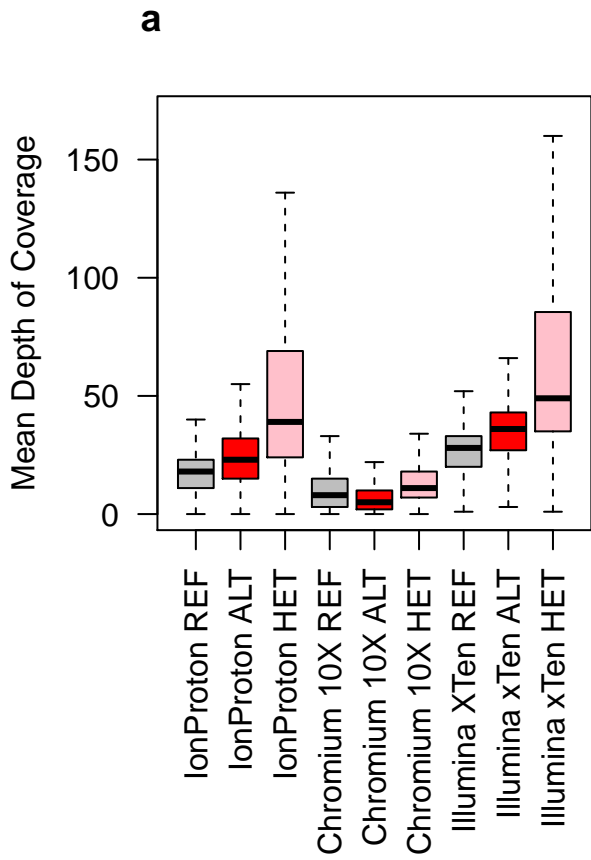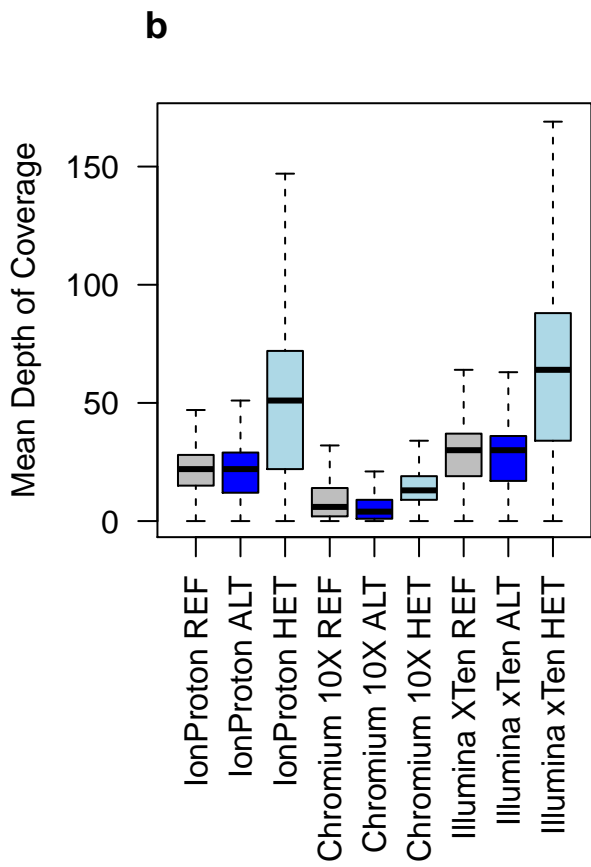

Supplement: Supplementary file 9 — Supplementary Figure 4. [file 41598_2021_92993_MOESM9_ESM.pdf]
